# Post-Mortem Analysis of Heroin Biomarkers, Morphine and Codeine in Stomach Wall Tissue in Heroin-Related Deaths

**DOI:** 10.3390/toxics10080473

**Published:** 2022-08-14

**Authors:** Ahmed I. Al-Asmari, Hassan Alharbi, Torki A. Zughaibi

**Affiliations:** 1 Laboratory Department, Ministry of Health, King Abdul-Aziz Hospital, P.O. Box 6470, Jeddah 21442, Saudi Arabia; 2 King Fahd Medical Research Center, King Abdulaziz University, P.O. Box 80216, Jeddah 21589, Saudi Arabia; 3 Poison Control and Forensic Chemistry Center, Ministry of Health, P.O. Box 21543, Jeddah 21176, Saudi Arabia; 4 Department of Medical Laboratory Sciences, Faculty of Applied Medical Sciences, King Abdulaziz University, P.O. Box 80216, Jeddah 21589, Saudi Arabia

**Keywords:** forensic toxicology, opiates, opioids, LC-MS/MS, postmortem, stomach wall tissue

## Abstract

Toxicological analysis of some cases can be complicated by poor sample quality caused by decomposition. Although heroin-related deaths have been researched extensively, the interpretation of toxicology findings in these cases is challenging, especially in instances where blood samples are unavailable. Thus, it is important to develop analytical methods for different sample types. In this study. a method for the quantification of 6-monoacetylmorphine, 6-acetylcodeine, morphine, and codeine in postmortem stomach wall tissue using liquid chromatography coupled with tandem mass spectrometry was developed and validated. All calibration curves prepared with the stomach wall tissue were linear and ranged from 0.5–1000 ng/g with determination coefficients of >0.99 and a lower limit of quantification of 1.0 ng/g. The coefficients of variation for within-run precision and between-run precision were <9%. Matrix effects of stomach wall tissues and their extraction recoveries were investigated and ranged from −19% to +17% and 76% to 80%, respectively. Among the 16 analyzed heroin-related death cases, 6-monoacetylmorphine, 6-acetylcodeine, morphine, and codeine were detected in 75%, 31%, 100%, and 94% of all stomach wall tissues with median concentrations of 90 ng/g, 20 ng/g, 140 ng/g, and 30 ng/g, respectively. This study provides new data on the distribution of 6-monoacetylmorphine, 6-Acetylcodeine, morphine, and codeine in postmortem stomach wall tissue and suggests the usefulness of alternative matrices for investigating heroin-related fatalities when blood samples are unavailable. In addition, the prevalence of 6-monoacetylmorphine in the stomach wall tissue was higher than that in the liver and kidney tissues.

## 1. Introduction

Heroin is known to be unstable in biological samples; thus, heroin biomarkers such as 6-monoacetylmorphine (6-MAM) and 6-acetylcodeine (6-AC) have been validated to prove the administration of heroin and to confirm that death occurred immediately following heroin ingestion if they are detected in blood samples. Within 2–5 min, heroin is converted to its active metabolite 6-MAM which is rapidly hydrolyzed to morphine. This morphine is conjugated to form polar metabolites that are excreted via urine [1,2]. 6-AC is a heroin biomarker that does not arise from heroin metabolism but from impurities introduced during the heroin manufacturing process [3,4]. 

Although heroin-related deaths have been researched extensively, the interpretation of toxicology findings is challenging, especially in instances where blood samples are unavailable [3,5]. Few studies have explored solid tissues for forensic toxicology analysis [6,7,8,9]. Solid tissues may be the only specimens available for testing in some cases. Therefore, the detection of heroin-related metabolites in these tissues is crucial for the successful toxicological interpretation of such cases [10].

Ratios between blood or other bodily fluids and tissue specimens have been published in many previous studies [1,6,8,10]. The results obtained from other sample types are not meant to replace blood results, as blood samples are considered the golden standard for postmortem forensic toxicology investigations, [6] but rather to aid with interpretation; especially in cases where blood is unavailable, as is the case with low blood volume (children’s cases), accidents, fire, putrefaction, and other circumstances [8,11]. In fact, each suspected drug-related fatality should be interpreted with care; obtaining more data and information surrounding the case will aid in better interpretation of the cases under investigation [6].

In practice, data interpretation is challenging because the source of the morphine can be illegally obtained heroin metabolites, poppy seed ingestion, or over-the-counter codeine-containing medication (as a metabolite), and morphine is also clinically used for pain management [8,12] In addition, poor sample quality due to trauma and decomposition complicates toxicological analytical methods in some cases [6,12,13]. At the Jeddah Poison Control Center and Forensic Medical Chemistry Center (JPCC), toxicological examination has on occasion been conducted on samples exhibiting advanced putrefaction. The hot weather that is prevalent year-round in Saudi Arabia complicates cases where the death occurred in desert areas. In three cases received at JPCC in 2014, there were no bodily fluids suitable for analysis. Alternative tissue samples such as liver tissue, kidney tissue, and stomach wall tissue were collected. In these cases, when stomach wall tissue samples were analyzed, high concentrations of 6-MAM and 6-AC were detected. Consequently, all stomach wall samples from other cases that yielded positive results for heroin and its biomarkers were collected and analyzed using described methods.

Few studies have evaluated stomach wall tissues in drug-related postmortem cases [6,14,15,16]. This may be due to the easy availability of other bodily fluid specimens that can be obtained in liquid form and the lack of data regarding stomach wall specimens in real postmortem cases. Chaturvedi and Rao reported the analysis of stomach tissues homogenized in water for toxicological analysis in a case of death related to self-administered fentanyl [15]. Studies evaluating the value of solid tissues such as liver, muscle, and kidney tissues as alternative specimens to blood in heroin-related deaths cases have been reported. Maskell et al. reported a procedure that employs solid phase extraction (SPE, using a Varian Bond Elut cartridge) and analysis of heroin-related metabolites from homogenized solid tissues (liver and muscle) [8]. In the study by Al-Asmari, Clean Screen^®^ cartridges were used for SPE of analytes from liver and kidney tissues [6]. In the study by Uboh et al., ß-glucuronidase was added to the homogenized and deproteinized tissues [17].

With putrefaction, it is hypothesized that stomach wall tissue is autolyzed faster compared to liver tissue, which makes liver tissue the preferred sample type for testing in such cases. To date, there is no protocol for the extraction of stomach tissue for heroin biomarkers is available. The mechanisms of trapping these analytes in tissues are scarce and poorly understood which limits their use as postmortem specimens. Although animal studies have been conducted with respect to heroin-related intoxication, they were found to be limited and/or unhelpful in obtaining the information required in this matter. For example, relative concentrations of morphine in human and animal studies are thought to be unhelpful due to the differences in their entero-hepatic metabolism [18,19].

**Figure 1:**
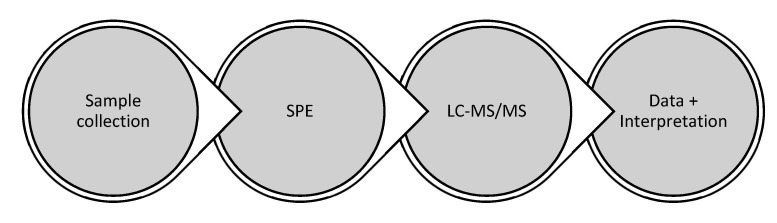
Graphical workflow for sample processing.

To the best of the authors’ knowledge, no previous scientific data from real heroin-related death cases demonstrated the applicability of stomach wall tissues as a specimen for heroin biomarkers, morphine and codeine. In this study, SPE and liquid chromatography with tandem mass spectrometry (LC-MS/MS) methods were developed and validated for the detection and quantification of 6-MAM, 6-AC, morphine, and codeine in postmortem stomach wall tissues, and this procedure was used in circumstances where extreme decomposition had rendered all bodily-fluids unavailable. A graphical workflow for the processing of samples is shown in Figure 1. This study is the first to report the distribution of heroin-related metabolites in postmortem stomach wall specimens. The purpose of this study was to examine and stress the value of stomach wall tissues; therefore, the aforementioned concentrations and results were compared to blood, gastric content, liver, and kidney results from our previously published reports [1,6], wherein the stomach wall tissues were tested in parallel for the same deceased to investigate the distribution of the heroin-related compound in these matrices in heroin-related fatalities postmortem cases. 

## 2. Materials and Methods

### 2.1. Chemicals and Reference Standards

All the solvents used in this study were high-performance liquid chromatography (HPLC) grade and were purchased from BDH (Poole, UK). Ammonium formate was obtained from Sigma Aldrich (Steinheim, Germany). Morphine, 6-MAM, codeine, 6-AC, and their corresponding internal standards were purchased from Lipomed (Arlesheim, Switzerland). SPE cartridges (Clean Screen^®^, CSDAU203) were obtained from United Chemical Technologies (Bristol, PA, USA). Stomach wall tissues were homogenized using Stomacher^®^ 400 and Stomacher 80 bags (dimensions: 101 × 152 mm with filter) purchased from Seward limited company (West Sussex, UK).

### 2.2. Case Samples

#### 2.2.1. Inclusion/Exclusion Criteria

In the current study, and in agreement with previous studies, cases attributed to heroin use were confirmed by certain criteria: the presence of heroin biomarkers (6-MAM) in blood or any of the tested specimens [3,5,19,20]. In addition, the ratio between morphine and codeine was higher than 1 [21,22,23]. Information during the autopsy was reviewed to confirm that no codeine medication was administered by reviewing crime scene location, police reports, forensic pathologist’s requests, or detection of other concomitant drugs such as acetaminophen (in which codeine is mostly available in a pharmaceutical preparation in combination with acetaminophen in Saudi Arabia). These served as the inclusion/exclusion criteria for including them in this study.

#### 2.2.2. Sample Collections

Blood samples were collected from subclavian in tubes containing 1% sodium fluoride (BNaF). For liver tissue, samples were collected from 3–5 sites across the deep right lobe to avoid contamination. Samples were taken from both kidneys (left and right) from the middle of the organs. The entire gastric contents retained at autopsy were utilized. Stomach wall tissue samples were collected from 3–5 sites of the stomach wall tissue (including mucosa). The specimens collected during the autopsy included blood in 11 cases (69%), gastric contents in 11 cases (69%), liver tissue in 15 cases (94%), kidneys tissue in 13 cases (81%), and stomach wall tissue in 16 cases (100%). 

#### 2.2.3. Data Collection 

As per JPCC policy, all submitted postmortem bodily fluid and tissues (including stomach wall tissue) should be quantified and reported. Therefore, all data presented in this report are available on the Forensic Toxicology Reports Jeddah (FTRJ) database and analyzed at the time of reporting without re-analyzing stomach wall tissue. 

#### 2.2.4. Toxicological Testing 

Toxicology testing for drugs and metabolites that are commonly encountered in postmortem toxicology in whole blood, urine, or tissue specimens, includes immunoassay screening, alcohol testing, carbon monoxide testing, general unknown screening (GUS) using gas chromatography-mass spectrometry, and LC-MS/MS for confirming all suspected positive results. GUS depended on the case, and target drugs and their metabolites in bodily fluids and solid tissues, for example, heroin, opioids, amphetamines, cocaine, benzodiazepine, barbiturate, antipsychotics, cannabinoids, and their metabolites were detected using adapted LC-MS/MS methods previously reported [24,25,26] while GUS for nontarget drugs were conducted using GC-MS [27,28].

A fully validated LC-MS/MS method was used for the analysis of 60 drugs and metabolites which are most encountered in postmortem-related cases. This includes, but is not limited to, heroin biomarkers, morphine, and codeine, as was reported with blood samples in a previous study [26]. The purpose of applying such methods was to attempt to establish a systematic toxicological analysis approach for all postmortem cases regardless of case circumstances, age, postmortem interval time (PMI), or history of drug use. In this approach, a larger number of suspected substances and their metabolites have been successfully screened with low sample volume, which helps eliminate any doubts regarding interference of other drugs and, more importantly, this provides valuable information to the pathologist in case multiple drugs were co-ingested. The use of this validated LC-MS/MS along with the GUS procedure aims to cover a larger number of suspected substances of abuse. In cases where the GUS identifies new substances not already included in the LC-MS/MS panel, special methods are then created to confirm and quantify them. 

#### 2.2.5. Stomach Wall Tissue Sample Preparation

In this study, stomach wall tissue was washed prior to weighing and homogenized using deionized water to limit contamination from gastric contents and surrounding fluids. Next, 5 g specimens were weighed and placed into a Stomacher bag to be homogenized; each tissue specimen was diluted 1:2 (*w/v*, using 1% NaF aqueous solution) and then homogenized for 5 min in the Stomacher^®^ 400. Then, 0.5 g of stomach wall tissue homogenate was placed into a 15 mL glass tube, and 50 µL of internal standard solution (50 ng/g, 6-MAM-d3, morphine-d_3_, and codeine-d_3_) was spiked into each tube, which was then vortexed and centrifuged at 3700× *g* for 10 min. 

#### 2.2.6. Preparation of Other Postmortem Matrices

BNaF and gastric content samples was prepared as previously described [1]. A sample of 1 mL BNaF or gastric contents was transferred after centrifugation into 15 mL glass tubes and spiked with 50 μL of the internal standard (50 µg/mL, 6-MAM-d3, morphine-d_3_, and codeine-d_3_), followed by the addition of 2 mL phosphate buffer (pH 6, 0.1 M). Liver and kidney tissues were prepared as previously reported [6]. In that report, the same sample preparation procedure that was used to prepare the stomach wall tissue samples was used to prepare both liver and kidney tissues for SPE.

#### 2.2.7. Solid-Phase Extraction 

A previously reported SPE procedure for heroin-related metabolite extraction from postmortem liver and kidneys tissues was used for stomach wall tissue sample analysis [6]. For this procedure, the SPE cartridge was first preconditioned with 2 mL methanol, deionized water, and phosphate buffer (0.1 M, pH 6), respectively. Then, the samples (BNaF, gastric contents, liver tissue, kidney tissue, and stomach wall tissue) were loaded onto the SPE column. Next, the SPE cartridges were washed twice using 1 mL deionized water, followed by 1 mL acetic acid (0.1 M), and a full vacuum was applied for 5 min to dry the SPE cartridges. Then, the SPE column was washed twice with 1 mL hexane, and a full vacuum was applied for 2 min. After that, fraction A was eluted using 3 mL hexane/ethyl acetate (1:1, *v/v*), and the elution tube was subsequently removed. Then, each SPE column was washed with 3 mL methanol and dried for 2 min with full vacuum. Fraction B was then eluted using 3 mL dichloromethane/isopropanol/ammonium hydroxide (78:20:2, *V/V/V*). Fractions A and B were combined, evaporated to dryness under a nitrogen stream, and reconstituted in 200 μL of the initial mobile phase; 1 μL of this sample was injected into the LC-MS-MS instrument.

### 2.3. Instrumentation

A previously reported LC-MS/MS procedure for heroin-related metabolites analysis from postmortem liver and kidney tissues was adapted for stomach wall tissue sample analysis [6]. The distribution of heroin-related metabolites included in this study was investigated using an ultra-high performance liquid chromatography obtained from Shimadzu (model: Shimadzu Nexera UHPLC system, Shimadzu, Kyoto, Japan) coupled with a Shimadzu triple quadrupole mass spectrometer using electrospray ionization (ESI) (model: LCMS-8050; Shimadzu, Kyoto, Japan). The chromatography separation was achieved using a Raptor Biophenyl column (50 × 3.0 mm, 2.7 μm, Restek, Bellefonte, PA, USA) attached to a Security Guard Raptor Biophenyl column (5.0 × 3.0 mm, 2.7 µm, Restek, USA). The column oven temperature was maintained at 40 °C during the analysis. In this study, a gradient elution consisting of 10 mM ammonium formate adjusted to pH 3 (A) and methanol (B) at a flow rate of 0.3 mL/min was applied for the separation of the analytes of interest. Gradient elution started with 3% (B) in the first minute of the run, followed by an increase in the B percentage to 5% at minute 2. Afterward, the B percentage was increased to 95% at minute 15, returning to the initial mobile 3% of B at minute 16, and it was maintained for 4 min. 

In this procedure, a positive ion mode using multiple reaction monitoring was employed. Each analyte of interest was tuned using 1 ppm of certified references standard and best conditions were recorded. Three different gases were used: air was used as a heating gas (flow: 10 L/min), nitrogen was used as a nebulizing (flow: 3 L/min), and a drying gas (flow: 15 L/min), and argon was used for collision-induced dissociation (CID). The interface, desolvation line (used to deliver the sample to the section under vacuum), and heat block temperatures were optimized, and the best conditions were determined to be 300 °C, 250 °C, and 400 °C, respectively. In addition to the retention time (RT), two ion transitions were monitored for each analyte and their deuterated internal standards; the first transition ion was used as a quantifier ion, whereas the second transition ion was used as the qualifier ion.

Deuterated internal standards for all analytes of interest were available and used except 6-AC, for which no deuterated internal standards were available commercially. In this study, we used codeine-d3 as the internal standard for both codeine and 6-AC. LC-MS/MS details for analytes of interest and their corresponding internal standards are listed in Table 1. Data were analyzed with Labsolution software (version 5.75, Shimadzu, Kyoto, Japan).

### 2.4. Method Validation

The method was fully validated in accordance with the forensic toxicology international guidelines [29,30]. All method validation parameters for BNaF, gastric content, liver, and kidney tissues have been previously published [1,6]. The current study focused on the validation and applicability of the described method in analyzing analytes of interest using stomach wall tissue. Stomach wall tissues obtained from a negative postmortem case were used as blank controls during validation. Blank stomach wall tissue specimens that were tested by described methods and confirmed to be negative for the analytes of interest were used for the entire method validation studies.

Heroin biomarkers and their metabolites were spiked into the stomach wall tissues at concentrations of 0.5, 1, 5, 10, 25, 50, 100, 250, 500, and 1000 ng/g (in 10 separate occasions, *n* = 5 for each concentration) to evaluate linearity. Three different controls having low, medium, and high concentrations (5, 100, and 800 ng/g, respectively) across the linear calibration curve were used to assess precision, accuracy, matrix effect, and recovery of the analytes of interest. Six different stomach wall tissues that tested negative by the described method were used to investigate the matrix effects and recovery experiments (*n* = 5 for each concentration). 

The sensitivity of the method was assessed by evaluating the limit of detection (LOD), limit of quantification (LOQ), and upper limit of quantification (ULOQ) according to the ANSI/ASB standard [30]. In that protocol, the LODs were calculated using the following equation: LOD = (3.3sy)/Avgm (where ‘sy’ is the standard deviation of the intercept and ‘Avgm’ is the average slope). The LOD values are included in Table 2. The LOQs were determined for each analyte of interest using three different stomach wall tissues that were spiked (1 ng/g); each sample was analyzed in triplicates in five different runs. The highest calibration point in the calibration curve (1000 ng/g) was set as ULOQ in this study.

Interferences from endogenous matrix materials in addition to the most common drug encountered in daily postmortem cases and their metabolites were evaluated. The most common drug examples are opiates, opioids, amphetamines, benzodiazepines, barbiturates, antipsychotics, cannabinoids, and cocaine. Ten stomach wall tissue specimens (obtained from post-mortem drug-free cases) were used for blank specimen assessment. Five of them were spiked with standards only (6-MAM, 6-AC, morphine, and codeine) and the other five specimens were spiked with internal standards only (6-MAM-d_3_, morphine-d_3_, and codeine-d_3_) (3 replicates were used for each sample). In addition, standards containing the most common drug encountered in daily postmortem cases were spiked into the stomach wall tissues at a concentration of 2000 ng/g and extracted using the described method before injection into the LC-MS/MS system. In addition, each batch of the samples included the following: blank stomach wall tissue without the addition of standards and internal standards, blank stomach wall tissue spiked with only internal standards at a concentration of 50 ng/g, and blank specimens spiked with both standards at a different concentration ranging from 0.5–1000 ng/mL and internal standards 50 ng/mL and were run as part of the calibration curve studies.

Carryover was assessed by the injection of the blank samples containing only the mobile phase following the analysis of the highest concentration in the calibration curve range (1000 ng/g). The point of testing carryover is to ensure that the high concentrations of the linear dynamic range (LDR, 0.5–1000 ng/g) have no effect on the results of the next sample, and to avoid any contamination from previous samples that have a higher concentration than that of the ULOQ (1000 ng/g). In this study, samples were run in duplicate and average concentration was then reported. Washing of the LC-MS/MS column was conducted with a high organic modifier (95% methanol) for at least 5 min after the last analytes appeared in the LC-chromatogram and prior to the next injection to ensure no contamination occurred from previous injections. Moreover, any samples found with concentrations higher than that of the ULOQ would be subjected to dilution to bring them to LDR before the results were reported. Dilution integrity was investigated by diluting the analytes of interest 50 and 100 times with deionized water followed by analyzing them (in 5 separate occasions, *n* = 3 for each sample). The method validation parameters included in the study are detailed in Table 2.

### 2.5. Statistical Analysis

Descriptive statistics are presented as frequency, percentage, mean, median, and range, whenever appropriate. The data were analyzed with Excel 2019 software (Microsoft, Redmond, WA, USA) and Statistical Packages for Software Sciences (SPSS) version 21 (Armonk, New York, IBM Corporation). Variables were compared using the Spearman correlation coefficient. A *p*-value < 0.05 was considered significant.

## 3. Results

### 3.1. Method Validation

A method for analyzing 6-MAM, 6-AC, morphine, and codeine in routine postmortem stomach wall tissues using LC-MS/MS was developed and validated; the validation was fit for the purpose. Method validation parameters including the study of linearity, sensitivity, precision, accuracy, selectivity, matrix effect, recovery, dilution integrity, and carryover results were deemed acceptable. As indicated in Table 2, linear calibration curves having coefficients of determination > 0.99 were obtained for 6-MAM, 6-AC, morphine, and codeine in stomach wall tissues. The lowest LOD values ranged between 0.2–0.4 ng/g, with an LOQ of 1.0 ng/g for all analytes of interest. There was no observed interference from drugs and their metabolites that were commonly detected in daily postmortem cases, blank stomach wall specimens, or carryover effects from the last injection.

### 3.2. Case Samples

**Figure 2:**
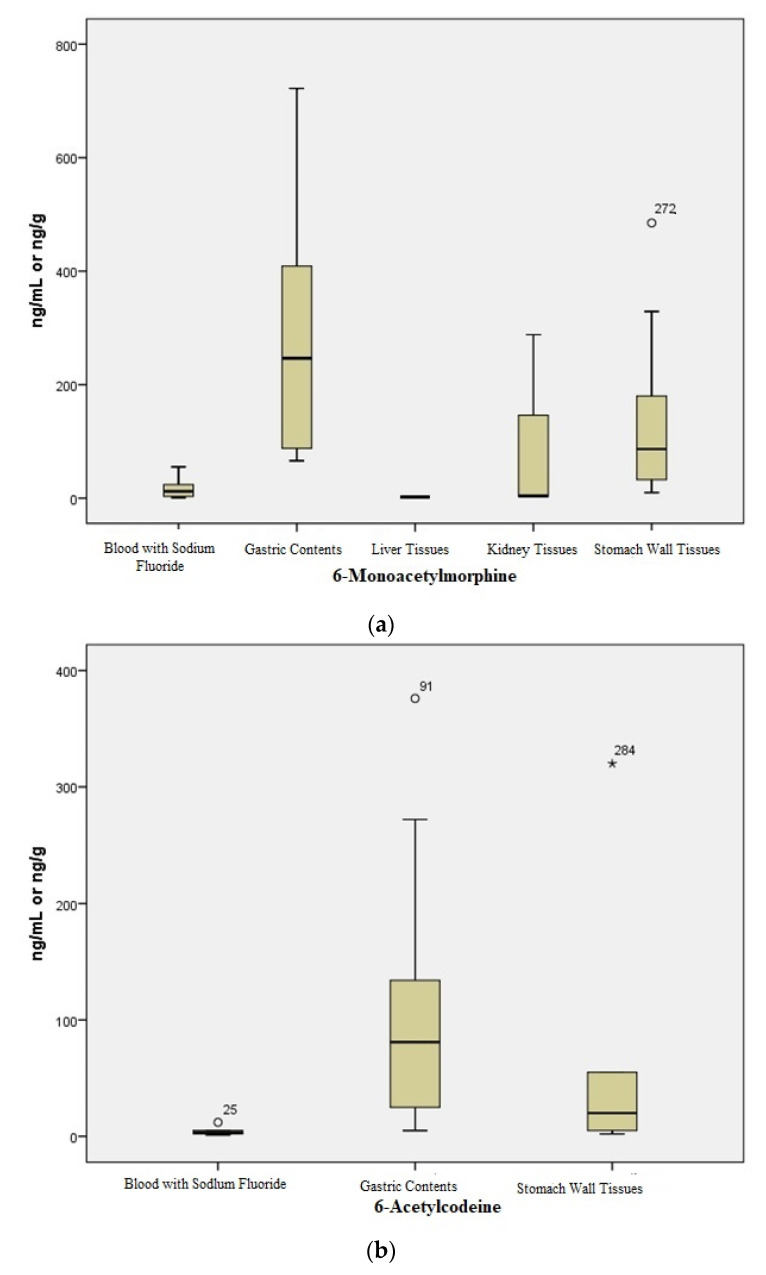

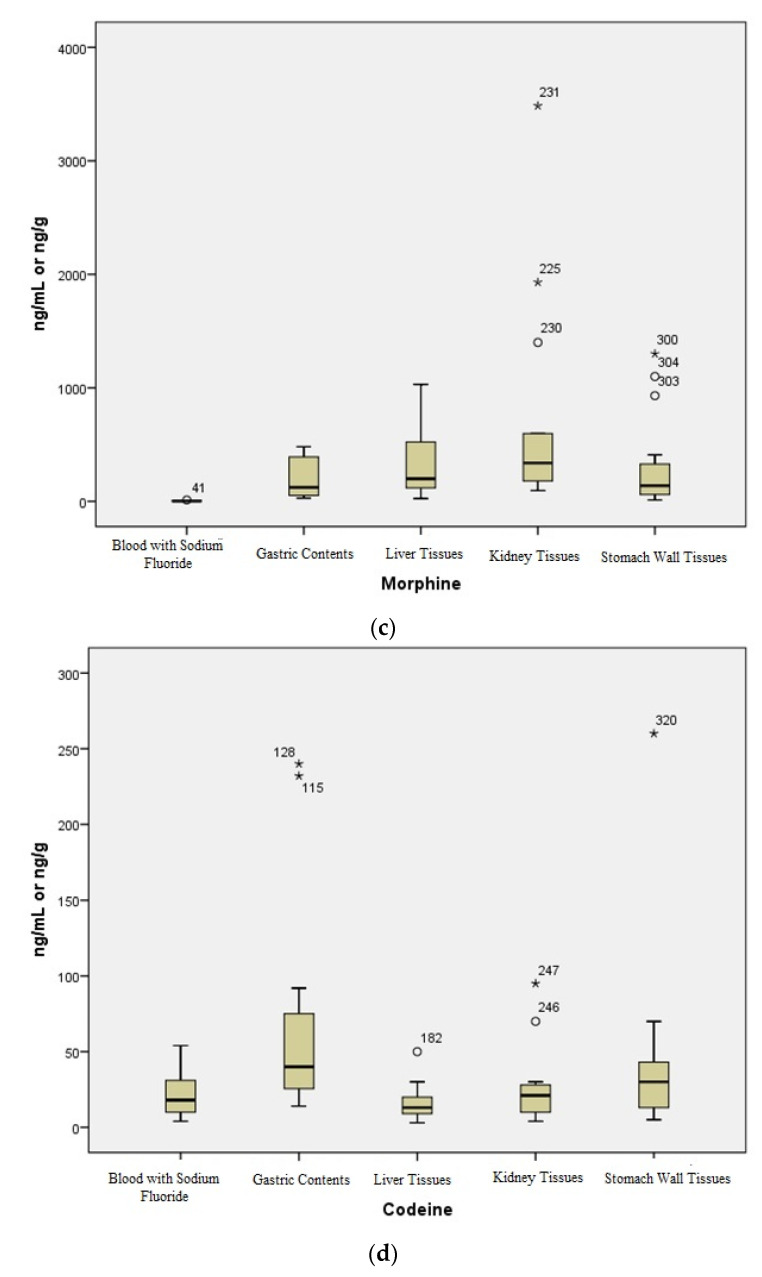
Concentration ratios of (**a**) 6-acetylmorphine (6-MAM), (**b**) 6-acetylcodeine (6-AC; no 6-AC was detected in liver and kidney tissues), (**c**) morphine, and (**d**) codeine in the blood with sodium fluoride (ng/mL), gastric contents (ng/mL), liver tissues (ng/g), kidney tissues (ng/g,) and stomach wall tissues (ng/g) of 16 heroin-related death cases included in the current study. The horizontal boxes represent the median concentration ratio, and the box lengths represent the 25–75th percentile. The whiskers represent the smallest and largest value within 1.5 times the interquartile range, and circles (outlier) represent values exceeding at least 1.5 times the interquartile range and extremes (asterisks) represent values exceeding at least 3.0 times the interquartile range.

Sixteen heroin-related death cases (all male) were analyzed in this study. In three cases, no bodily fluids were available for testing. In this investigation, the mean age of the decedent was 32 years (median: 33, range: 21–70 years). The postmortem time interval between death and sample collection ranged from 24 to 240 h (median, 108 h). The heroin-related deaths case details, including, history, age, body condition (putrefaction), and other co-ingested drugs are described in Table 3, and heroin-related metabolite concentration details are listed in Table 4 and visualized in Figure 2.

6-MAM was detected in 12 out of the 16 (75%) stomach wall tissue samples included in this study. A longer PMI time (range, 72–240 h) was observed in the four cases where 6-MAM was not detected. Codeine was quantified in 15 stomach wall tissues (94%), and 6-AC was found to be positive in 5 cases (31%). The median free morphine to free codeine ratio in stomach wall tissues was 7.0 (range: 1.0–24, *n* = 15). The median free morphine concentration in the stomach wall was 1.0-fold (range: 0.2–13) that in BNaF.

The Spearman correlation (Rs) analysis was used to determine the relationship between 6-MAM, 6-AC, morphine, and codeine in BNaF, gastric contents, liver, kidneys, and stomach wall tissue specimens (Table 5). There were negative to weak positive correlations in the 6-MAM concentration between BNaF/stomach wall tissues, BNaF/gastric contents, and gastric contents/stomach wall tissues 6-MAM: Rs = −0.510 [*p*-value = 0.2], 0.070 [*p*-value = 0.8] and Rs = 0.200 [*p*-value = 0.7], respectively. In contrast, there were positive correlations in the 6-AC concentrations; however, these correlations were not considered statistically significant: Rs = −0.600 [*p*-value = 0.3], 0.820 [*p*-value = 0.1], and Rs = 0.700 [*p*-value = 0.2], respectively, even though only a few samples tested positive for 6-AC. 6-MAM and 6-AC were either negative or positive, and samples were not adequate to compare liver and kidney tissues. 

In this study, the highest morphine concentration was found in kidneys tissues (median: 340 ng/g), followed by liver tissues (median: 200 ng/g), BNaF (median: 180 ng/mL), stomach wall tissues (median: 140 ng/g), and gastric contents (median: 120 ng/mL). A weak positive correlation in morphine concentrations was observed between BNaF/stomach wall tissues (Rs = 0.040 [*p*-value = 0.9]), BNaF/gastric contents (Rs = 0.200 [*p*-value = 0.6]). In contrast, a strong positive correlation and statistically significant relationship were observed between gastric contents/stomach wall tissues (Rs = 0.900 [*p*-value = 0.04]); a strong correlation was observed with codeine between BNaF and other specimen types, gastric contents, stomach wall tissues, liver tissues, and kidneys tissues with Rs = 0.670 [*p*-value = 0.03], Rs = 0.711 [*p*-value = 0.02], Rs = 0.700 [*p*-value = 0.03], and Rs = 0.711 [*p*-value = 0.02], respectively. 

When liver and kidneys tissues were available for testing, the correlations between morphine and codeine concentrations were Rs = −0.300 [*p*-value = 0.4] and Rs = 0.664 [*p*-value = 0.05] (gastric contents/liver); Rs = −0.242 [*p*-value = 0.5] and Rs = 0.633 [*p*-value = 0.06] (gastric contents/kidneys); Rs = 0.200 [*p*-value = 0.5] and Rs = 0.500 [*p*-value = 0.1] (stomach wall tissues/liver), Rs = 0.210 [*p*-value = 0.5] and Rs = 0.610 [*p*-value = 0.03] (stomach wall tissues/kidneys tissues), respectively. In addition, the correlations between morphine and codeine concentrations were often positive and statistically significant in all specimen types except gastric contents, which revealed a weakly positive and not statistically significant correlation, as indicated in Table 5. A good correlation was observed between liver tissue/kidney tissues, with Rs = 0.760 [*p*-value = 0.004] for morphine and Rs = 0.884 [*p*-value = 0.0001] for codeine (Table 5).

## 4. Discussion

In this study, the distribution of heroin biomarkers in stomach wall tissue was evaluated. Owing to the lack of previous studies on heroin biomarker concentrations in stomach wall tissues, the results obtained in this study were compared to other results from solid tissues, such as liver tissues and kidney tissues. A recent study (involving 31 cases) presented the distribution of 6-MAM, 6-AC, morphine, and codeine in liver and kidney tissues; few cases were positive for 6-MAM (liver, four cases; kidney, two cases) and all tissues were negative for 6-AC [6]. In their study, poor non-statistically significant correlations were reported in terms of morphine concentration between liver tissue/BNaF and kidney tissue/BNaF with Rs values of 0.270 [*p*-value = 0.2] and 0.350 [*p*-value = 0.1], respectively. In contrast, Maskell et al. reported 44 heroin-related fatalities and investigated the correlation of femoral blood with liver by calculating Rs. Their findings showed a strong correlation and statistically significant relationship between liver tissues/blood with Rs = 0.860 [*p*-value = 2 × 10^−2^]. Same observation was reported by Duflou et al., with Rs = 0.610 [*p*-value = 0.003] [19]. This disagreement between the findings can be explained by poor sample quality due to postmortem changes in the current study. 

Notably, in the current study, 6-MAM was detected in stomach wall tissues in the majority of cases (75%), even in cases with heavy putrefaction, while 6-AC was measurable in 31% of cases. 6-AC appeared to be more stable in gastric content samples than in stomach wall tissue samples; 6-MAM, morphine, and codeine showed comparable concentrations in these two tissue types in most cases.

Although gastric contents can be used to qualitatively assess the source of opioids, this method has limitations; this possibly explains the low number of studies on this sample type in the literature [1,19,31]. Gastric contents are rarely homogeneous, and thus accurate drug concentrations cannot be obtained from this sample type. Gastric content sample volumes received for analysis are often much larger than those needed for chromatography analysis (often I mL), which affects the accuracy of the estimated concentration [3]. In contrast, stomach wall tissues can be precisely weighed and homogenized to quantify analytes in a small number of specimens. 

Solid tissue samples with severe decomposition, were sent for forensic toxicological analysis. Among them, liver tissue homogenate is most tested for illegal drugs in alternative biological specimens [7,9,32]. Morphine in the liver is always high, sometimes explained by the fact that the liver is considered a likely site of morphine deposition when postmortem redistribution occurs [6]. The liver persists in the body long after the stomach wall tissue, and therefore, it is of interest to compare the current results of stomach wall tissues with those of liver tissues from the same deceased individuals. In this study, a good correlation was observed in morphine and codeine concentrations between the liver, kidney, and stomach wall tissues. The advantage of stomach wall tissues over liver and kidney tissues is the detectability of heroin biomarkers (6-MAM and 6-AC) despite long PMIs. It was reported that liver specimens are not useful for 6-MAM to identify a heroin user since the primary site for 6-MAM metabolism to morphine occurs in the liver [7]. This correlation of the results of the liver along with the stomach wall tissues strengthens the findings of the current work. 

Studies evaluating the value of gastric contents in heroin-related deaths attempt to distinguish between injected and oral heroin overdose [1,16,19]. However, the morphine concentration in gastric contents is not sufficient for such analyses as morphine from the gastric contents most likely undergoes entero-hepatic circulation [19]. Previous reports found that higher concentrations of morphine were detected in gastric contents than that in the blood in heroin-injected cases [16,19]. In addition, in two previous reports, all gastric contents were positive for heroin biomarkers, and therefore assist in attributing the source and cause of death to heroin use [1,31]. Duflou et al. [19] reported a statistically significant correlation in total morphine concentration between blood and gastric contents (Rs = 0.670; [*p*-value = 0.001]). In this study, a poor correlation in free morphine concentration was observed between these two matrices. In this study and previous reports [10,19], the free morphine concentrations in gastric contents were always higher than those in blood. 6-MAM was measurable in all gastric content samples in most previous studies [1,16,31], while 6-AC was quantified in gastric contents in one previous report [1]. In the current investigation, four of 16 cases (25%) tested negative for 6-MAM in stomach wall tissue. In these cases, a longer PMI time and heavy sample decomposition were observed. Codeine derived from 6-AC was present in most gastric content samples, in agreement with a previous report [1].

In the current study, heroin biomarker concentration in stomach wall tissues were hypothesized to be comparable to those in gastric contents. As heroin diffuses through stomach tissues into the surrounding blood and tissues, several factors may affect its concentration after death and decomposition. Heroin metabolites such as morphine conjugates are converted to free morphine by hydrolysis/degradation due to high temperature and low pH [33]. Moriya and Hashimoto stated that morphine conjugates are unstable and are converted to free morphine completely within 10 days when stored at 18–37 °C, while free morphine is stable in blood and tissues at 4.0 to 37.0 °C [16]. Similarly, Al-Asmari reported that the morphine concentration in the liver was 4 times higher than that in blood; the deconjugating of morphine glucuronides most likely occurs in internal solid tissues during decomposition [6]. Maskell et al., [8] found that morphine and its glucuronides were affected by postmortem redistribution (PMR). This was thought to be similar in the case of stomach wall tissues. In the current investigation, it must be stressed that PMI competes with PMR, as during the PMI period, degradation, and formation of analytes of interest or new compounds are formed, as is the case with ethanol production after death by microorganisms [4,34]. PMR is a term describing the increase or decrease in the concentration of the same analytes during the postmortem period [8]. The role of PMI in the stability of heroin biomarkers is a well-known phenomenon that should be taken into consideration when dealing with heroin-related fatalities [13,35]. In addition, it has been suggested that an increase in PMI can affect the concentration of heroin biomarkers, morphine, and codeine as a result of PMR [36]. It has been clearly observed in this study that the probability of detection of 6-MAM and 6-AC decreases with an increase in PMI. Although PMI and PMR are important factors for interpreting the source of opiates used and the cause of death, it is rarely discussed in previous reports. Gerostamoulos and Drummer [37] studied 40 heroin-related deaths with an average PMI of 59 h. They found no significant difference between antemortem and postmortem samples of morphine and its metabolites. The same conclusion was observed by Fugelstad et al.; they believed postmortem changes after deaths have less effect on heroin-related metabolites and those analyte concentrations detected during autopsy reflected concentrations at the time of death [38]. Maskell et al. recommended the use of vitreous fluid to confirm heroin use, with minimal morphine change observed in their study [39].

It can be seen clearly in this study that the concentration of morphine in stomach wall tissues was lower than in BNaF and kidney specimens. The median morphine concentrations in kidney were almost double that of stomach wall tissues, with a median of 0.4-fold (*n* = 13), respectively, while the median morphine concentration in gastric contents and was almost similar to that of stomach wall tissues. This can be interpreted by the fact that heroin biomarkers were stable in stomach wall tissue and gastric contents for a longer period compared to that in blood, liver tissues, and kidney tissues [19]. Kidneys are known to have a high affinity for free morphine as compared to its glucuronide [3,6,16]. The liver is the organ responsible for free morphine metabolizing morphine conjugates, which can lead to a higher concentration of morphine glucuronides compared to that of free morphine. However, it has been reported that morphine concentration in the liver was four times higher than that in blood; the deconjugating of morphine glucuronides most likely occurs as part of postmortem changes after death in internal solid tissues during decomposition [6,16]. Al-Asmari and Anderson studied the stability of 6-MAM, 6-AC, morphine, and codeine under different storage conditions, in blood and without preservatives. In that study, a decrease in both heroin biomarkers was observed and in contrast, a 45% and 48% increase in morphine and codeine was observed [22].

The case was different when we compared heroin biomarkers and codeine ratios between stomach wall tissues and other specimens; the median codeine ratios were considered higher in stomach wall tissues than BNaF (1.3-fold, *n* = 11), liver 1.3-fold (*n* = 13), and kidneys (1.6-fold, *n* = 12). However, a higher codeine concentration was observed in gastric contents compared to that of stomach wall tissues with a median ratio of 3-fold (*n* = 10). The higher concentration in gastric contents for both morphine and codeine compared to that of stomach wall tissues may have occurred because they undergo cycles of entero-hepatic circulation which resulted in a significant increase in morphine and codeine in gastric contents after morphine and codeine glucuronides deconjugated in the liver [19]. Duflou et al. mentioned that high opioid concentrations in gastric contents might have an effect of ion trapping, where the compounds could have crossed the gastric intestinal mucosa and were unable to return to the bloodstream. After death, the body relaxes, increasing the permeability of the intestinal walls, and bound drugs are released from storage depots [19]. The differences in concentrations of analytes of interest between gastric contents and stomach wall tissues in the current study provide evidence that stomach wall tissue is an independent matrix, is unique, and has its own benefits as postmortem specimens. Contamination from gastric contents is an issue that cannot be excluded, however, both matrices can be used as complementary specimens to confirm drug intake when blood or other body fluid samples are unavailable. 

Interestingly, the median 6-MAM ratios of gastric content were found to be higher than that of stomach wall tissues; in contrast, 6-MAM was detected in fewer kidney and liver tissues. Both liver and kidneys are found to be not suitable for 6-MAM or 6-AC testing as reported in previous reports [6,7,16,40]. Moriya and Hashimoto [16] concluded that gastric contents and urine are important keys to solving heroin-related cases due to their high rate of 6-MAM detection. In this study, and in agreement with Moriya and Hashimoto’s study, it was concluded that stomach wall tissues and gastric contents should be considered as samples of choice in case no blood or bodily fluids are available in deaths involving heroin, not only due to their higher detection rate of 6-MAM but their higher detection rate of 6-AC as well. To the best of the authors’ knowledge, this is the first time that 6-MAM and 6-AC were detected in stomach wall tissues and gastric contents in parallel. One limitation of the current investigation is not including other heroin biomarkers such as papaverine and noscapine as these biomarkers are not fully validated in JPCC and more research is needed to study their role in heroin-related deaths. In previous reports, tested illicit heroin samples were negative for papaverine and noscapine. However, these compounds can be present in over-the-counter drugs and food products containing poppy seeds. Therefore, papaverine and noscapine found in specimens cannot be used as evidence of illicit heroin consumption [8].

The interpretations of stomach wall tissue and other solid alternative specimen results are considered complicated owing to many limitations, such as a lack of information in the literature for comparisons, and because these solid matrices are not considered an alternative to traditional samples such as blood, urine, and vitreous humor. Instead, they are complementary specimens to these bodily fluid results. However, with advanced putrefaction, the choice of sample is limited to solid tissues in which results obtained from these solid matrices can be useful in conducting the postmortem toxicological investigation [6,19]. 

## 5. Conclusions

Solid tissues may be the only specimens available for postmortem toxicological analysis in cases with advanced putrefaction of the dead body. Using stomach wall tissue and other solid alternative specimens helped determine the source of opioids administered. However, in case no traditional post-mortem samples (such as blood, urine, and vitreous humor) are available, interpreting the concentrations of analytes of interest in these specimens is still complicated due to several limitations, including the lack of previous studies to draw comparisons. Instead, the concentration of heroin biomarkers in stomach wall tissues is proven to be a good complement to bodily fluid results. The uniqueness of this study is the full optimization and validation of target analytes in stomach wall tissue specimens. This method has been applied to postmortem analysis of heroin-related cases for about five years and has yielded reproducible and accurate results. To the best of the author’s knowledge, this is the first study to evaluate 6-MAM, 6-AC, morphine, and codeine distribution in stomach wall tissues. This study provides novel insights and adds to the limited data on heroin biomarkers in postmortem tissues. In conclusion, samples for post-mortem analysis in cases with a longer PMI show significant decomposition; analysis of free morphine in solid tissue specimens is more reliable, convenient, cost-efficient, and time-efficient than analyzing morphine and its glucuronides. In this study, the highest morphine concentration was found in kidney tissues, followed by liver tissues, BNaF, stomach wall tissues, and gastric contents. In this study, a higher detection rate of 6-MAM was observed in the stomach wall tissues compared to other solid tissues (liver and kidney tissues). These findings suggest the usefulness of alternative matrices for investigating heroin-related fatalities when blood samples are unavailable.

## Figures and Tables

**Table 1:** LC-MS/MS data for heroin biomarkers, morphine, and codeine.

Analytes ^&^	Internal Standards	Retention Time (min)	Quantifier	Qualifier
6-MAM	6-MAM-d3	6.85	*m/z* = 328–165	*m/z* = 328–221
6-MAM-d3 ^#^	-	6.95	*m/z* = 331–165	*m/z* = 331–221
6-AC	Codeine-d3	9.3	*m/z* = 342–225	*m/z* = 342–165
Morphine	Morphine-d3	4.7	*m/z* = 286–165	*m/z* = 286–153
Morphine-d3 ^#^	-	4.6	*m/z* = 289–165	*m/z* = 289–153
Codeine	Codeine-d3	6.8	*m/z* = 300–165	*m/z* = 300–44
Codeine-d3 ^#^	-	6.7	*m/z* = 303–165	*m/z* = 300–199

& Analytes: 6-Monoacetylmorphine (6-MAM), 6-Acetylcodeine (6-AC), # Internal standard.

**Table 2:** LC-MS/MS data for heroin biomarkers, morphine, and codeine.

			Analytes			
			6-Monoacetylmorphine	Morphine	6-Acetylcodeine	Codeine
Linearity (10 different days, 5 replicate/control)		R^2^ *	0.999	0.999	0.999	0.999
		LDR ^Φ^ (ng/g)	0.5–1000.0	0.5–1000.0	0.5–1000.0	0.5–1000.0
		Intercept	0.4488028	−0.0063087	−0.044456	0.0756881
		STDEV ^#^ (intercept)	0.0036603	0.000405	0.001627	0.0006525
		Slope	0.045315	0.005116	0.013986	0.013706
Sensitivity (ng/g)		Limit of detection	0.3	0.3	0.4	0.2
		Limit of quantification	1.0	1.0	1.0	1.0
		Upper limit of quantification	1000.0	1000.0	1000.0	1000.0
Accuracy and precision	Low control (5 ng/g)	Mean (ng/g)	5.0	5.1	5.1	4.9
		STDEV	0.2	0.3	0.4	0.3
		%E ^Ҿ^	−1	3	2	−3
		Within-run%CV **	5	5	8	3
		Between-run%CV	5	7	7	6
	Medium Control (100 ng/g)	Mean (ng/g)	96.1	100.9	103.1	101.0
		%E	−4	1	3	1
		Within Run%CV	4	4	5	5
		Between Run%CV	6	5	8	7
	High control (800 ng/g)	Mean (ng/g)	790.3	811.4	808.0	789.8
		%E	−1	1	1	−1
		Within Run%CV	2	2	5	1
		Between Run%CV	2	3	4	2
Dilution	Dilution low control ^Ψ^ (250 ng/g)	Mean (ng/g)	4.8	5.2	4.8	4.8
		%E	−2	2	−2	−2
		Within-run%CV	3	6	5	5
		Between-run%CV	4	7	4	5
	Dilution High control ^¥^ (7500 ng/g)	Mean (ng/g)	76.8	73.2	70.5	74.3
		%E	2	−2	−6	−1
		Within-run%CV	7	9	5	5
		Between-run%CV	9	9	9	7
Matrix effects (%)	5 ng/g	Mean (ng/g)	−19	14	−20	17
		%CV	6	11	7	13
Recovery (%)		Mean (ng/g)	78	80	76	79
		%CV	10	5	4	2
Matrix effects (%)	100 ng/g	Mean (ng/g)	−15	8	−8	4
		%CV	12	7	4	8
Recovery (%)		Mean (ng/g)	82	84	82	91
		%CV	3	5	6	4
Matrix effects (%)	800 ng/g	Mean (ng/g)	−5	2	−4	2
		%CV	6	3	3	5
Recovery (%)		Mean (ng/g)	94	92	94	94
		%CV	7	5	8	5

* R^2^: Coefficient of determination; ^Φ^ LDR: Linear dynamic range; # STDEV: Standard deviation; CV: coefficient of variation; **^Ҿ^** %E: Relative error.^Ψ^: Dilution factor: 1:50 times; ^¥^ Dilution factor: 1:100 times.

**Table 3:** Demographic profiles of 16 cases included in this study.

Case no.	History	Age	Putrefaction	Other Drugs	
1.	The deceased was found dead in his home	44	None	None	
2.	The deceased found dead under a bridge, and the deceased was a heroin abuser	29	Heavy	Amphetamine	BNaF *: 91 ng/mL
3.	The deceased died inside a house; drug use was suspected, as there were needles and empty plastic bags next to the body	56	Heavy	None	
4.	The deceased was found dead in a desert area	31	Heavy	None	
5.	The deceased was found dead at home	70	Some	None	
6.	The deceased had a history of mental illness and drug abuse	26	None	None	
7.	The deceased was found dead in a car with no traces of violence or wounds. The deceased had a history of heroin addiction	46	Some	Alprazolam	BNaF: 30 ng/mL
				11-nor-Delta-9-Tetrahydrocannabinol (THC-COOH)	BNaF: 12 ng/mL
8.	The body of the deceased was found in a desert land with traces of acupuncture	25	None	None	
9.	The body was found in the downtown area, and was in a highly decomposed condition	33	Heavy	None	
10.	The body of the deceased was found in a desert land with traces of acupuncture	30	None	Amphetamine	BNaF: 450 ng/mL ST-W&: 845 ng/g Liver: 400 ng/g Kidneys: 800 ng/g
11.	Syringes and needles were found next to the body	43	Some	None	
12.	The deceased died with two people in a house. Drugs were found in the house	23	None	Cocaine	BNaF: 0.5 ng/mL Gastric content: 5 ng/mL
				Benzoylecgonine	BNaF: 5 ng/mL Liver: 30 ng/g
				Ecgonine methyl ester	BNaF: 19 ng/mL Gastric contents: 43 ng/mL ST-W: 25 ng/g Liver: 20 ng/g Kidney 30 ng/g
				Delta-9-Tetrahydrocannabinol (THC)	BNaF: 11 ng/mL Gastric contents: 121 ng/mL ST-W; 5 ng/g
				11-hydroxy-delta-9-THC (THC-OH)	BNaF: 3 ng/mL
				THC-COOH	BNaF: 17 ng/mL ST-W; 6 ng/g Liver: 40 ng/g Kidneys: 50 ng/g
				Alprazolam,	BNaF: 29 ng/mL Gastric contents: 780 ng/mL ST-W; 115 ng/g Liver: 55 ng/g Kidneys: 25 ng/g
				Pregabalin	BNaF: 610 ng/mL Gastric contents: 14,000 ng/mL ST-W; 1900 ng/g Liver: 1020 ng/g Kidneys: 1150 ng/g
13.	The deceased died with two people in a house. Drugs were found in the house	23	None	THC	BNaF: 20 ng/mL Gastric contents: 170 ng/mL
				THC-OH	BNaF: 8 ng/mL
				THC-COOH	BNaF: 92 ng/mL Gastric contents: 170 ng/mL ST-W; 30 ng/g Liver: 90 ng/g Kidneys: 60 ng/g
				Alprazolam,	BNaF: 43 ng/mL Gastric contents: 62 ng/mL ST-W; 15 ng/g Liver: 7 ng/g Kidneys: 3 ng/g
				Tramadol	BNaF: 57 ng/mL Gastric contents: 112 ng/mL ST-W; 150 ng/g Liver: 145 ng/g Kidneys: 140 ng/g
14.	The deceased died with two people in a house. Drugs were found in the house	21	None	THC	BNaF: 10 ng/mL Gastric contents: 5 ng/mL Liver: 5 ng/g
				THC-OH	BNaF: 4 ng/mL
				THC-COOH	BNaF: 18 ng/mL Gastric contents: 25 ng/mL Liver: 55 ng/g Kidneys: 60 ng/g
				Alprazolam,	BNaF: 2 ng/mL Gastric contents: 1 ng/mL Liver: 6 ng/g Kidneys: 3 ng/g
				Tramadol	BNaF: 40 ng/mL Gastric contents: 37 ng/mL ST-W; 160 ng/g Liver: 95 ng/g Kidneys: 135 ng/g
15.	The deceased was found dead in a car in the front passenger seat	45	Some	Ethanol	BNaF: 162 mg/dL Gastric contents: 350 mg/dL ST-W; 3.52 mg/g Liver: 6.15 mg/g Kidneys: 4.30 mg/g
				Ethyl glucuronide	BNaF: 14,300 ng/mL
				Ethyl sulfate	BNaF: 4100 ng/mL
				Amphetamine	BNaF: 310 ng/mL Gastric contents: 550 ng/mL ST-W; 380 ng/g Liver: 210 ng/g Kidneys: 160 ng/g
16.	The deceased was found dead at home with several sizes of syringes next to him. Death as a result of a heroin overdose	35	Some	None	

**Table 4:** Analyte concentrations in 16 heroin-related deaths analyzed in this study.

	Blood with Sodium Fluoride				Gastric Contents				Stomach Wall Tissues				Liver Tissues				Kidneys Tissues			
	(ng/mL)				(ng/mL)				(ng/g)				(ng/g)				(ng/g)			
Case no.	6-MAM ^ϕ^	6-AC ^λ^	MOR ^Ҿ^	COD ^Ҕ^	6-MAM	6-AC	MOR	COD	6-MAM	6-AC	MOR	COD	6-MAM	6-AC	MOR	COD	6-MAM	6-AC	MOR	COD
1	n.a *	n.a	n.a	n.a	n.a	n.a	n.a	n.a	84	neg ^#^	247	16	neg	neg	755	n.a.	n.a.	n.a.	n.a.	n.a.
2	n.a	n.a	n.a	n.a	397	11	72	39	35	2	75	30	neg	neg	80	10	neg	neg	95	9
3	n.a	n.a	n.a	n.a	n.a	n.a	n.a	n.a	138	neg	931	39	neg	neg	1031	28	4	neg	598	28
4	n.a	n.a	n.a	n.a	n.a	n.a	n.a	n.a	170	neg	160	24	neg	neg	650	17	neg	neg	535	21
5	25	neg	78	5	328	65	29	41	329	neg	29	41	n.a.	n.a.	n.a.	n.a.	288	neg	1930	19
6	5	neg	16	13	409	272	70	232	30	neg	11	9	2	neg	167		n.a.	n.a.	n.a.	n.a.
7	neg	neg	205	20	neg	neg	366	58	neg	neg	66	18	neg	neg	268	13	n.a.	n.a.	n.a.	n.a.
8	12	neg	467	23	165	97	29	40	89	neg	115		neg	neg	810	50	neg	neg	1400	70
9	17	neg	715	44	n.a	n.a	n.a	n.a	neg	neg	410	55	neg	neg	395	18	neg	neg	3485	95
10	neg	neg	172	16	n.a	n.a	n.a	n.a	neg	neg	230	45	neg	neg	200	9	neg	neg	270	25
11	55	12	26	4	66	25	122	14	neg	neg	50	5	neg	neg	120	6	neg	neg	110	8
12	1	5	102	10	88	44	34	20	15	neg	55	5	neg	neg	160	10	neg	neg	250	10
13	3	3	286	39	661	376	440	92	60	55	240	35	neg	neg	300	30	neg	neg	338	30
14	1	1	98	10	105	134	481	25	190	320	1300	70	neg	neg	80	10	2	neg	505	20
15	24	2	196	22	66	5	252	26	10	20	85	10	neg	neg	25	3	neg	neg	180	4
16	neg	neg	255	54	722	103	416	240	485	5	1100	260	neg	neg	118	20	neg	neg	140	26
Mean	15.9	4.6	218.0	21.7	300.7	113.2	210.1	75.2	136.3	80.4	319.0	44.1	n.a.	n.a.	343.9	16.9	98.0	n.a.	756.6	28.1
Median	12.0	3.0	184.0	18.0	246.5	81.0	122.0	40.0	86.5	20.0	137.5	30.0	n.a.	n.a.	200.0	13.0	4.0	n.a.	338.0	21.0
STDEV	17.4	4.4	200.7	16.0	245.2	121.2	183.8	82.3	143.3	135.6	411.6	62.7	n.a.	n.a.	315.1	12.9	164.5	n.a.	983.6	26.0
Min	1.0	1.0	16.0	4.0	66.0	5.0	29.0	14.0	10.0	2.0	11.0	5.0	n.a.	n.a.	25.0	3.0	2.0	n.a.	95.0	4.0
Max	55.0	12.0	715.0	54.0	722.0	376.0	481.0	240.0	485.0	320.0	1300.0	260.0	n.a.	n.a.	1031.0	50.0	288.0	n.a.	3485.0	95.0
6-MAM ϕ: 6-Monoacetylmorphine; 6-AC λ: 6-Acetylcodeine; MOR Ҿ: Morphine; COD Ҕ: Codeine; STDEV, standard deviation; n.a *: Sample not available; neg #: Negative.																				

**Table 5:** Correlation (Spearman-Rho [*p*-value]) between the heroin biomarkers and their metabolites concentration detected in 16 heroin-related postmortem cases in different postmortem specimens in the current study.

Specimens		Blood with Sodium Fluoride				Gastric Contents				Stomach Wall Tissues				Liver Tissues				Kidneys Tissues			
	Analyes ^&^	6-MAM	6-AC	MOR	COD	6-MAM	6-AC	MOR	COD	6-MAM	6-AC	MOR	COD	6-MAM	6-AC	MOR	COD	6-MAM	6-AC	MOR	COD
Blood with sodium fluoride	6-MAM	1																			
	6-AC	0.6 ** [0.3]	1																		
	MOR	−0.240 [0.6]	−0.3 [0.6]	1																	
	COD	−0.350 [0.4]	−0.451 [0.4]	0.872 [0.0002] *	1																
Gastric Contents	6-MAM	−0.300 [0.5]	−0.6 [0.3]	0.251 [0.5]	0.621 [0.07]	1															
	6-AC	−0.510 [0.2]	−0.6 [0.3]	0.120 [0.8]	0.451 [0.2]	0.650 [0.04] *	1														
	MOR	−0.350 [0.4]	−0.800 [0.1]	0.200 [0.6]	0.341 [0.3]	0.130 [0.7]	0.300 [0.4]	1													
	COD	−0.050 [0.9]	(−0.6) [0.3]	0.300 [0.4]	0.670 [0.03] *	0.900 [0.0004] *	0.600 [0.07]	0.140 [0.7]	1												
Stomach wall tissues	6-MAM	0.071 [0.8]	−0.400 [0.6]	0.200 [0.7]	0.200 [0.7]	0.520 [0.2]	0.400 [0.3]	0.100 [0.8]	0.470 [0.2]	1											
	6-AC	n.a.	0.820 [0.09]	0.300 [0.6]	0.100 [0.9]	0.500 [0.4]	0.600 [0.3]	0.900 [0.04] *	−0.500 [0.4]	0.200 [0.7]	1										
	MOR	−0.45 [0.2]	−0.900 [0.03] *	0.040 [0.9]	0.537 [0.9]	0.200 [0.6]	0.300 [0.5]	0.700 [0.03] *	0.500 [0.1]	0.531 [0.08]	0.700 [0.2]	1									
	COD	−0.3 [0.5]	−0.872 [0.05]	0.350 [0.3]	0.711 [0.02] *	0.600 [0.09]	0.400 [0.3]	0.100 [0.8]	0.668 [0.03]	0.863 [0.0006] *	0.200 [0.7]	0.702 [0.004] *	1								
Liver tissues	6-MAM	n.a.	n.a	n.a	n.a	n.a	n.a	n.a	n.a	n.a	n.a	n.a	n.a	1							
	6-AC	n.a.	n.a.	n.a.	n.a.	n.a.	n.a.	n.a.	n.a.	n.a.	n.a.	n.a.	n.a.	n.a	1						
	MOR	−0.050 [0.9]	0.5 [0.4]	0.200 [0.6]	0.200 [0.6]	0.352 [0.4]	0.600 [0.08]	−0.300 [0.4]	0.360 [0.3]	0.300 [0.4]	0.100 [0.9]	0.200 [0.5]	0.100 [0.8]	n.a.	n.a	1					
	COD	−0.381 [0.4]	−0.050 [0.9]	0.800 [0.01] *	0.700 [0.03] *	0.650 [0.08]	0.800 [0.01] *	0.100 [0.8]	0.664 [0.05]	0.420 [0.3]	0.300 [0.6]	0.500 [0.09]	0.500 [0.1]	n.a.	n.a.	0.864 [0.0001] *	1				
Kidneys tissues	6-MAM	n.a.	n.a.	n.a.	n.a.	n.a.	n.a.	n.a.	n.a.	n.a.	n.a.	n.a.	n.a.	n.a.	n.a.	n.a	n.a	1			
	6-AC	n.a.	n.a.	n.a.	n.a.	n.a.	n.a.	n.a.	n.a.	n.a.	n.a.	n.a.	n.a.	n.a.	n.a.	n.a.	n.a.	n.a	1		
	MOR	0.120 [0.8]	−0.700 [0.2]	0.433 [0.2]	0.150 [0.7]	0.050 [0.9]	0.520 [0.2]	−0.242 [0.5]	0.250 [0.5]	0.381 [0.3]	0.700 [0.2]	0.210 [0.5]	0.381 [0.2]	n.a.	n.a.	0.760 [0.004] *	0.700 [0.02] *	n.a.	n.a	1	
	COD	0.340 [0.4]	−0.200 [0.7]	0.700 [0.04] *	0.711 [0.02] *	0.644 [0.06]	0.870 [0.003] *	0.050 [0.9]	0.633 [0.06]	0.400 [0.3]	0.300 [0.6]	0.630 [0.02] *	0.610 [0.03]	n.a.	n.a.	0.742 [0.005] *	0.884 [0.0001] *	n.a.	n.a.	0.650 [0.1] *	1

^&^ Analytes: 6-monoacetylmorphine (6-MAM), 6-acetylcodeine (6-AC), morphine (MOR) and codeine (COD), ^#^ n.a.: Negative samples for analytes of interest or not enough positive sample to make comparison. ** Spearman-Rho [*p*-value] * Correlation is considered statically significant [*p*-value <0.05].

## Data Availability

The data underlying this article will be shared on reasonable request to the corresponding author.

## References

[1] Al-Asmari A.I. (2020). Postmortem Fluid Concentrations of Heroin Biomarkers and Their Metabolites. J. Forensic Sci..

[2] Skopp G. (2004). Preanalytic Aspects in Postmortem Toxicology. Forensic Sci. Int..

[3] Moffat A.C., Osselton M.D., Widdop B. (2011). Clarke’s Analysis of Drugs and Poisons in Pharmaceuticals, Body Fluids and Postmortem Material.

[4] Skopp G. (2010). Postmortem Toxicology. Forensic Sci. Med. Pathol..

[5] Randall B. (2008). Disposition of Toxic Drugs and Chemicals in Man.

[6] Al-Asmari A.I. (2020). Postmortem Liver and Kidney Tissue Concentrations of Heroin Biomarkers and Their Metabolites in Heroin-Related Fatalities. J. Forensic Sci..

[7] Felby S., Christensen H., Lund A. (1974). Morphine Concentrations in Blood and Organs in Cases of Fatal Poisoning. Forensic Sci..

[8] Maskell P.D., Wilson N.E., Seetohul L.N., Crichton M.L., Beer L.J., Drummond G., de Paoli G. (2019). Postmortem Tissue Distribution of Morphine and Its Metabolites in a Series of Heroin-Related Deaths. Drug Test. Anal..

[9] Margalho C., Franco J., Corte-Real F., Vieira D.N. (2011). Illicit Drugs in Alternative Biological Specimens: A Case Report. J. Forensic Leg. Med..

[10] Thaulow C.H., Øiestad Å.M.L., Rogde S., Karinen R., Brochmann G.W., Andersen J.M., Høiseth G., Handal M., Mørland J., Arnestad M. (2018). Metabolites of Heroin in Several Different Post-Mortem Matrices. J. Anal. Toxicol..

[11] Vandenbosch M., Pajk S., van den Bogaert W., Wuestenbergs J., van de Voorde W., Cuypers E. (2021). Post-Mortem Analysis of Opioids and Metabolites in Skeletal Tissue. J. Anal. Toxicol..

[12] Thaulow C.H., Øiestad Å.M.L., Rogde S., Andersen J.M., Høiseth G., Handal M., Mørland J., Vindenes V. (2018). Can Measurements of Heroin Metabolites in Post-Mortem Matrices Other than Peripheral Blood Indicate If Death Was Rapid or Delayed?. Forensic Sci. Int..

[13] Thiblin I., Eksborg S., Petersson A., Fugelstad A., Rajs J. (2004). Fatal Intoxication as a Consequence of Intranasal Administration (Snorting) or Pulmonary Inhalation (Smoking) of Heroin. Forensic Sci. Int..

[14] Havig S.M., Vindenes V., Øiestad Å.M.L., Rogde S., Thaulow C.H. (2021). Methadone, Buprenorphine, Oxycodone, Fentanyl and Tramadol in Multiple Postmortem Matrices. J. Anal. Toxicol..

[15] Chaturvedi A.K., Rao N.G.S., Baird J.R. (1990). A Death Due to Self-Administered Fentanyl. J. Anal. Toxicol..

[16] Moriya F., Hashimoto Y. (1997). Distribution of Free and Conjugated Morphine in Body Fluids and Tissues in a Fatal Heroin Overdose: Is Conjugated Morphine Stable in Postmortem Specimens?. J. Forensic Sci..

[17] Uboh C.E., Rudy J.A., Railing F.A., Enright J.M., Shoemaker J.M., Kahler M.C., Shellenberger J.M., Kemecsei Z., Das D.N., Soma L.R. (1995). Postmortem Tissue Samples: An Alternative to Urine and Blood for Drug Analysis in Racehorses. J. Anal. Toxicol..

[18] Levine W.G. (1978). Biliary Excretion of Drugs and Other Xenobiotics. Annu. Rev. Pharmacol. Toxicol..

[19] Duflou J., Darke S., Easson J. (2009). Morphine Concentrations in Stomach Contents of Intravenous Opioid Overdose Deaths. J. Forensic Sci..

[20] Jones A.W., Holmgren A., Ahlner J. (2012). Concentrations of Free-Morphine in Peripheral Blood after Recent Use of Heroin in Overdose Deaths and in Apprehended Drivers. Forensic Sci. Int..

[21] Jones A.W., Holmgren A. (2011). Concentration Ratios of Free-Morphine to Free-Codeine in Femoral Blood in Heroin-Related Poisoning Deaths. Leg. Med..

[22] Al-Asmari A.I., Anderson R.A. (2007). Method for Quantification of Opioids and Their Metabolites in Autopsy Blood by Liquid Chromatography-Tandem Mass Spectrometry. J. Anal. Toxicol..

[23] Jakobsson G., Truver M.T., Wrobel S.A., Gréen H., Kronstrand R. (2021). Heroin-Related Compounds and Metabolic Ratios in Postmortem Samples Using LC-MS-MS. J. Anal. Toxicol..

[24] Bidny S., Gago K., Chung P., Albertyn D., Pasin D. (2017). Simultaneous Screening and Quantification of Basic, Neutral and Acidic Drugs in Blood Using UPLC-QTOF-MS. J. Anal. Toxicol..

[25] Di Rago M., Saar E., Rodda L.N., Turfus S., Kotsos A., Gerostamoulos D., Drummer O.H. (2014). Fast Targeted Analysis of 132 Acidic and Neutral Drugs and Poisons in Whole Blood Using LC-MS/MS. Forensic Sci. Int..

[26] Al-Asmari A.I. (2020). Method for the Identification and Quantification of Sixty Drugs and Their Metabolites in Postmortem Whole Blood Using Liquid Chromatography Tandem Mass Spectrometry. Forensic Sci. Int..

[27] Maurer H.H. (2008). Chapter 12 Forensic Screening with GC-MS. Handb. Anal. Sep..

[28] Wylie F.M., Torrance H., Seymour A., Buttress S., Oliver J.S. (2005). Drugs in Oral Fluid: Part II. Investigation of Drugs in Drivers. Forensic Sci. Int..

[29] Peters F.T., Drummer O.H., Musshoff F. (2007). Validation of New Methods. Forensic Sci. Int..

[30] (2019). American Academy of Forensic Sciences Standards Board 2019. ANSI/ASB Standard 036; Method Validation in Forensic Toxicology.

[31] DeRienz R.T., Baker D.D., Kelly N.E., Mullins A.M., Barnett R.Y., Hobbs J.M., Daniels J.A., Harshbarger K.E., Ortiz A.M. (2018). Child Fatalities Due to Heroin/Fentanyl Exposure: What the Case History Missed. J. Anal. Toxicol..

[32] Wyman J., Bultman S. (2004). Postmortem Distribution of Heroin Metabolites in Femoral Blood, Liver, Cerebrospinal Fluid, and Vitreous Humor. J. Anal. Toxicol..

[33] Crandall C.S., Kerrigan S., Aguero R.L., LaValley J., McKinney P.E. (2006). The Influence of Collection Site and Methods on Postmortem Morphine Concentrations in a Porcine Model. J. Anal. Toxicol..

[34] Al-Asmari A.I., Altowairgi M.M., Al-Amoudi D.H. (2022). Effects of Postmortem Interval, Putrefaction, Diabetes, and Location of Death on the Analysis of Ethyl Glucuronide and Ethyl Sulfate as Ethanol Biomarkers of Antemortem Alcohol Consumption. Forensic Sci. Int..

[35] Butzbach D.M. (2010). The Influence of Putrefaction and Sample Storage on Post-Mortem Toxicology Results. Forensic Sci. Med. Pathol..

[36] Rees K.A., Pounder D.J., Osselton M.D. (2013). Distribution of Opiates in Femoral Blood and Vitreous Humour in Heroin/Morphine-Related Deaths. Forensic Sci. Int..

[37] Gerostamoulos J., Drummer O.H. (2000). Postmortem Redistribution of Morphine and Its Metabolites. J. Forensic Sci..

[38] Fugelstad A., Ahlner J., Brandt L., Ceder G., Eksborg S., Rajs J., Beck O. (2003). Use of Morphine and 6-Monoacetylmorphine in Blood for the Evaluation of Possible Risk Factors for Sudden Death in 192 Heroin Users. Addiction.

[39] Maskell P.D., Albeishy M., de Paoli G., Wilson N.E., Seetohul L.N. (2016). Postmortem Redistribution of the Heroin Metabolites Morphine and Morphine-3-Glucuronide in Rabbits over 24 h. Int. J. Leg. Med..

[40] Goldberger B.A., Cone E.J., Grant T.M., Levine B.S., Smialek J.E. (1994). Disposition of Heroin and Its Metabolites in Heroin-Related Deaths. J. Anal. Toxicol..

